# Supplementary material for: Establishing a surveillance system on sexual and reproductive health and rights (SRHR) of key populations (KPs) at risk of compromised outcome of SRHR- A protocol for a mixed-method study
Source: PLoS One. 2023 Jul 27;18(7):e0289010. doi: 10.1371/journal.pone.0289010 (PMC10374132; doi:10.1371/journal.pone.0289010)
Supplement: S1 File — (DOCX) [file pone.0289010.s001.docx]

**Annex 1: Site selection for SRHR surveillance for KPs**

| **KPs** | **Target sample size** | **Size estimation data of KPs by district** | | | | |
| --- | --- | --- | --- | --- | --- | --- |
|  |  | **Jashore** | **Khulna** | **Chattogram** | **Sylhet** | **Dhaka** |
| **FSW** | 513 | 3,506 | 3,442 | 4,048 | 4,886 | 19,294 |
| **MSM** | 566 | 494 | 1,796 | 7,756 | 3,219 | 9,379 |
| **MSW** | 551 | 456 | 697 | 1,897 | 660 | 3,031 |
| **TGW (hijra)** | 610 | 44 | 273 | 673 | 522 | 1,485 |
| **Total KP** | **2,240** | **4,500** | **6,208** | **14,374** | **9,287** | **33,189** |
|  | **Comparison: Target sample size vs. size estimation data** | | | | | |
| **FSW** | 513 | √ | √ | √ | √ | √ |
| **MSM** | 566 | X | √ | √ | √ | √ |
| **MSW** | 551 | X | √ | √ | √ | √ |
| **TGW (hijra)** | 610 | X | X | X | X | √ |

Note: ‘√’ refers to target sample size is less than size estimation data, otherwise ‘X’

**Annex 2: SRHR indicators to be measured among KPs**

| **SL#** | **Indicators** | **KPs** | | | |
| --- | --- | --- | --- | --- | --- |
|  |  | **FSW** | **MSM** | **MSW** | **Hijra** |
|  |  | **Indicator applicability** | | | |
|  | **Indicators to be measured at the Individual level** | | | | |
|  | **Family Planning and Contraception** |  |  |  |  |
| 1 | Percentage of individuals who currently use any contraceptive method | √ | √ (married) | √ (married) |  |
| 2 | Percentage of individuals who currently use any modern contraceptive method (such as male and female sterilization, pill, IUD, injectables, im-plants and male condom) | √ | √ (married) | √ (married) |  |
| 3 | Percentage of individuals who decided to use family planning, alone or jointly with their husband/wife/other sex partners | √ | √ (married) | √ (married) |  |
|  | **Sexual and Reproductive Health** |  |  |  |  |
| 4 | Prevalence of HPV* | √ | √ | √ | √ |
| 5 | Prevalence of Syphilis (active) | √ | √ | √ | √ |
| 6 | Prevalence of Neisseria Gonorrhoea (NG)** | √ | √ | √ | √ |
| 7 | Prevalence of Chlamydia Trachomatis (CT)** | √ | √ | √ | √ |
| 8 | Comprehensive knowledge of HIV^§^ | √ | √ | √ | √ |
| 9 | Used condom in the last sex act with a male sex partner | √ | √ | √ | √ |
| 10 | Used condom in the last sex act with a female sex partner |  | √ | √ |  |
| 11 | Percentage of obstetric and gynaecological hospital admissions for different causes | √ |  |  |  |
| 12 | Percentage of women of reproductive age at risk of pregnancy who report trying for a pregnancy for two years or more | √ |  |  |  |
| 13 | Percentage of births that are reported as unintended | √ |  |  |  |
| 14 | Percentage of populations have knowledge on SRHR | √ | √ | √ | √ |
| 15 | Percentage of individuals took steroids/hormone/surgery to address their gender dysphoria |  |  | √ | √ |
| 16 | Percentage of individuals received SRH services to manage adverse effects of steroids/hormone use | √ |  | √ | √ |
| 17 | Percentage of individuals received psychosexual and psychosocial counselling services (ICT based/face to face) | √ | √ | √ | √ |
| 18 | Percentage of individuals received management of STI | √ | √ | √ | √ |
| 19 | Percentage of methamphetamine users uptake counselling services | √ | √ | √ | √ |
| 20 | Percentage of individuals that suffered sexual and reproductive health concerns (e.g., erectile dysfunction, premature ejaculation, loss of libido, etc.) |  | √ | √ |  |
| 21 | Percentage of individuals received respectful care and human rights in provision of SRH information and services at public and private except NGO facilities | √ | √ | √ | √ |
| 22 | Number of individuals who have experienced adverse childhood experiences (e.g. sexual abuse) | √ | √ | √ | √ |
|  | **Maternal and New-born Health Services** |  |  |  |  |
| 23 | Percentage of women attended at least four antenatal care visits during last pregnancy | √ |  |  |  |
| 24 | Percentage of all births attended by skilled birth attendant | √ |  |  |  |
| 25 | Percentage of women age 15-19 who have begun childbearing | √ |  |  |  |
|  | **Indicators to be measured at the Facility level (Service availability and readiness)** |  |  |  |  |
|  | **Family Planning and Contraception** |  |  |  |  |
| 26 | Percentage of primary service delivery points with at least 3 modern methods of contraception available on the day of the assessment | √ | √ | √ |  |
| 27 | Utilisation of essential SRHR services in public/private/NGO health facilities | √ | √ | √ | √ |
| 28 | Number of health care service providers trained in essential SRHR services | √ | √ | √ | √ |
|  | **Sexual and reproductive health services** |  |  |  |  |
| 29 | Availability, readiness and quality of STIs management services | √ | √ | √ | √ |
| 30 | Availability, readiness and quality of screening cervical and HPV | √ | √ | √ | √ |
| 31 | Availability, readiness and quality of other essential SRH services | √ | √ | √ | √ |
|  | **Maternal and New born Health Services** |  |  |  |  |
| 32 | Utilization of maternal and new-born services in public/private/NGO health facilities | √ |  |  |  |
| 33 | Availability, readiness and quality of basic and comprehensive obstetric care (haemorrhage, eclampsia, PAC etc.) | √ |  |  |  |
|  | **Indicators to be measured at the National and Policy level** |  |  |  |  |
|  | **Advocacy and Public Engagement** |  |  |  |  |
| 34 | Number of national laws, policies and strategies relating to SRHR implemented or strengthened through GAC-funded projects | √ | √ | √ | √ |
| 35 | Whether universal access to contraceptive and SRH information and services is included in national policy | √ | √ | √ | √ |
| 36 | Number of advocacy and public engagement activities completed by GAC-funded partners which are focused on SRHR | √ | √ | √ | √ |
|  | **Covid-19 related: Indicators to be measured at the Individual level** |  |  |  |  |
| 37 | Percentage of individuals have knowledge on Covid-19 transmission and prevention | √ | √ | √ | √ |
| 38 | Percentage of individuals infected with Covid-19 during lifetime | √ | √ | √ | √ |
| 39 | Percentage of KPs received vaccination for Covid-19 during last 12 months | √ | √ | √ | √ |

* HPV will be determined for MSW and hijra from anorectal and oropharyngeal samples, for FSW, from cervical swab samples and for MSM, from urine samples (detail is provided in the laboratory methods)

** NG and CT will be determined for MSW and hijra from anorectal and oropharyngeal samples, for FSW, from cervical swab samples and for MSM, from urine samples (detail is provided in the laboratory methods)

*^§^* This indicator will be computed by correct answers to five questions [1]:

1. Can people reduce their risk of HIV by using a condom correctly and consistently in any type of sex?
2. Can people reduce their risk of HIV by avoiding sex with multiple partners?
3. Can a person get HIV through mosquito bite?
4. Can a person get HIV by sharing a meal with someone who is HIV infected?
5. Can you tell by looking at someone whether s/he is infected with HIV?

**Annex 3: Sample sizes in each KP at the baseline**

| **SL#** | **Indicators** | **KPs** | | | |
| --- | --- | --- | --- | --- | --- |
|  |  | **FSW** | **MSM** | **MSW** | **Hijra** |
|  |  | **Values of the indicators (%)** | | | |
|  | ***Serological*** |  |  |  |  |
| 1. | Prevalence of active syphilis | 1.5 [2] | 13.8 [2] | 13.8 [2] | 12.8 [2] |
|  | ***Biological*** |  |  |  |  |
| 2 | Prevalence of HPV (Anal) |  |  | HPV 16: 20.3  HPV 18: 10.5  HPV 6: 20.4  [3, 4] | HPV 16: 8  HPV 6: 6  [5] |
|  | Prevalence of HPV (Cervical) | HPV 16: 10.1  HPV 18: 5.4  HPV 6: 3.6  [3] |  |  |  |
| 3. | Prevalence of Neisseria gonorrhoea (NG) | - 1. (Cervical) [6] |  | 4.4 (anal),  10.6 (oral)  [7] | 0.6 (anal),  10.7 (oral)  [7] |
| 4. | Prevalence of Chlamydia trachomatis (CT) | 6.3 (Cervical) [6] |  | 1.8 (anal),  0.9 (oral)  [7] | 1.2 (anal),  1.2 (oral)  [7] |
|  | ***Behavioural*** |  |  |  |  |
| 5. | Comprehensive knowledge of HIV^§^ | 26.4 [8] | 35.5 [8] | 37.0 [8] | 35.3 [8] |
| 6. | Used condom in the last sex act with a male sex partner | 78.7 [8] | 54.0 [8] | 53.5 [8] | 41.1 [8] |
| 7. | Percentage of FSW attended at least four antenatal care visits during pregnancy | 27.7 [9] |  |  |  |
| 8. | Percentage of births from FSW attended by skilled birth attendant | 54.1 [9] |  |  |  |
| 9. | Percentage of births from FSW that are reported as unintended | 26.8 [10] |  |  |  |
|  |  | **Calculated sample sizes** | | | |
|  | ***Biological*** |  |  |  |  |
| 1. | Prevalence of active syphilis | 357 | 430 | 394 | 230 |
| 2. | Prevalence of HPV (Anal) |  |  | 348  405  350 | 375  305 |
|  | Prevalence of HPV (Cervical) | 398  313  456 |  |  |  |
| 3. | Prevalence of Neisseria gonorrhoea (NG) | 313 |  | 259,  407 | **610**  362 |
| 4. | Prevalence of Chlamydia trachomatis (CT) | 356 |  | 413,  **551** | 377  377 |
|  | ***Behavioural*** |  |  |  |  |
| 5. | Comprehensive knowledge of HIV | 414 | 524 | 479 | 408 |
| 6. | Used condom in the last sex act with a male sex partner | 363 | **566** | 506 | 425 |
| 7. | Percentage of FSW attended at least four antenatal care visits during pregnancy | 425 |  |  |  |
| 8. | Percentage of births from FSW attended by skilled birth attendant | **513** |  |  |  |
| 9. | Percentage of births from FSW that are reported as unintended | 418 |  |  |  |
| **Final sample size (taking the maximum in each KP)** | | **513** | **566** | **551** | **610** |
| **Grand total** | | **2,240** | | | |

# **LITERATURE CITED**

1. UNAIDS. Guidelines on construction of core indicators. Geneva, Switzerland UNAIDS. 2009.
2. ASP. Integrated Biological and Behavioural Surveillance for Key Populations at Risk of HIV. Dhaka, Bangladesh: AIDS/STD Control Programme (ASP), Ministry of Health and Family Welfare, 2020.
3. Farahmand M, Moghoofei M, Dorost A, et al. Prevalence and genotype distribution of genital human papillomavirus infection in female sex workers in the world: a systematic review and meta-analysis. BMC Public Health. 2020; 20: 1455.
4. Müller, E. E., Rebe, K., Chirwa, T. F., Struthers, H., McIntyre, J., & Lewis, D. A. (2016). The prevalence of human papillomavirus infections and associated risk factors in men-who-have-sex-with-men in Cape Town, South Africa. *BMC Infectious Diseases, 16*(1), 1-14.
5. Cranston, R. D., Carballo-Diéguez, A., Gundacker, H., Richardson, B. A., Giguere, R., Dolezal, C., . . . Piper, J. M. (2019). Prevalence and determinants of anal human papillomavirus infection in men who have sex with men and transgender women. *International journal of STD & AIDS, 30*(2), 154-162.
6. Khanam, R., Reza, M., Ahmed, D., Rahman, M., Alam, M. S., Sultana, S., Alam, A., Khan, S. I., Mayer, K. H. and Azim, T. (2017). "Sexually Transmitted Infections and Associated Risk Factors Among Street-Based and Residence-Based Female Sex Workers in Dhaka, Bangladesh." Sexually Transmitted Diseases 44(1): 22-29.
7. Khanam, R., Ahmed, D., Alam, M.S., Reza, M. Ashraf, L., Alam, A., Das, N., Ahmed, S., Rahman, M., Khan, S. I., Rana, A. K. M. M., Amin, M., Faruque, M. O., Mayer, K., Azim, T. Sexually Transmitted Infections among Male and Female Sex Workers, Females who Inject Drugs and Hijras under the Global Fund Project in Dhaka. Global Fund Rolling Continuation Channel Project of icddr,b. Final report 2015 (unpublished).
8. ASP. End Line Survey (Behaviour) on Continuation of the Prioritized HIV Prevention Services among Key Population in Bangladesh. Dhaka, Bangladesh: National AIDS/STD Control Program (ASP), Ministry of Health and Family Welfare, 2017.
9. Wahed, T., Alam, A., Sultana, S., Alam, N., & Somrongthong, R. (2017). Sexual and reproductive health behaviors of female sex workers in Dhaka, Bangladesh. *PloS one, 12*(4), e0174540.
10. Ampt, F. H., Willenberg, L., Agius, P. A., Chersich, M., Luchters, S., & Lim, M. S. (2018). Incidence of unintended pregnancy among female sex workers in low-income and middle-income countries: a systematic review and meta-analysis. *BMJ open, 8*(9), e021779.

**Annex 4: Relationship of objectives, outcome variables and method of measurement**

| **Objectives** | **Outcome variables** | **Method of measurement** |
| --- | --- | --- |
| 1. To estimate the prevalence of selected SRHR indicators (biological, behavioral and socio-demographics) | Biological:   1. Prevalence of active syphilis 2. Prevalence of HPV (high risk and low risk subtypes) 3. Prevalence of NG and CT (oral and anal)   Behavioural:   1. Knowledge of SRHR and HIV 2. Currently use any modern contraceptive method to limit child bearing 3. Used condom during last sex act with non-transactional and transactional male/hijra/female sex partners to prevent HIV and STIs 4. Decision making on condom during last sex act (alone or jointly) 5. Decision making on family planning methods (alone or jointly) 6. Received services to manage adverse effects of using steroids/hormones 7. FSWs attended at least four antenatal care visits during last pregnancy 8. Births in FSW attended by skilled birth attendant 9. Births in FSW that are reported as unintended 10. Knowledge on Covid-19 transmission and prevention | Quantitative survey.  The results of biological and behavioural indicators will be expressed in-terms of percentage points with 95% confidence intervals.  The categorical variables of the socio-demographics will be expressed by percentage points along with 95% confidence interval. Numerical variables will be expressed by mean along with standard deviation (if normally distributed) or by median along with inter-quartile range (IQR) (if not normally distributed). |
| 1. To identify emerging SRHR health problems among KPs to generate priority areas for research and interventions. | Any of the outcome variables mentioned against objective 1. | Quantitative survey.  Indicators will be expressed by percentage points/mean values/medians. |
| 1. To assess study participants’ knowledge on transmission and prevention of COVID-19 and impact of COVID-19 in the utilization of SRHR services. | 1. Participants had correct knowledge of transmission and prevention of COVID-19 2. Participants received SRHR services from any health facility (government/private/NGO) | Quantitative survey.  Correct knowledge of transmission and prevention of COVID-19 will be expressed by percentage points.  Cross-tabulations will be done among the participants who had correct knowledge of transmission and prevention of COVID-19 (yes/no) with the SRHR services from any health facility (government/private/NGO) (yes/no). Chi-square statistics will be used to assess the association. |
| 1. Generate evidence-based recommendations and conduct policy dialogue for interventions on SRHR with KPs. | Not applicable | Data from quantitative survey will be used to generate recommendations. In addition, qualitative methods such as in-depth interviews will be used to know what the participants want. Key informant interviews will also be used to know what the service providers and policy makers think in regards to the SRHR situation among the study population groups that will be used in formulating recommendations from the study. |
