# Supplementary material for: Establishing a surveillance system on sexual and reproductive health and rights (SRHR) of key populations (KPs) at risk of compromised outcome of SRHR- A protocol for a mixed-method study
Source: PLoS One. 2023 Jul 27;18(7):e0289010. doi: 10.1371/journal.pone.0289010 (PMC10374132; doi:10.1371/journal.pone.0289010)
Supplement: Attachment — Submitted filename: Response to the Reviewers Comments.docx [file pone.0289010.s002.docx]

**Response to the Reviewers’ Comments**

**Journal requirements:**

When submitting your revision, we need you to address these additional requirements.

1. Please ensure that your manuscript meets PLOS ONE's style requirements, including those for file naming. The PLOS ONE style templates can be found at

https://journals.plos.org/plosone/s/file?id=wjVg/PLOSOne_formatting_sample_main_body.pdf and

https://journals.plos.org/plosone/s/file?id=ba62/PLOSOne_formatting_sample_title_authors_affiliations.pdf

Response: In the first submission, by mistake, the font style of the manuscript was in Calibri with font size 11. However, this time we have made it into Times New Roman with 12 font size and therefore, the line number increased.

2. You indicated that you had ethical approval for your study. In your Methods section, please ensure you have also stated whether you will obtain consent from parents or guardians of the minors who will be included in the study or whether the research ethics committee or IRB specifically waived the need for their consent.

Response: Informed written assent will be taken from the participants of 15 to less than 18 years of age and informed written consent will be taken from those who will be 18 years or above years of age. Written assent will be taken from local gatekeepers (such as, hawkers in the spots or someone who belongs to the community of the participant and at the same time knows the participant very well and older in age, at least 18 years, than the participant) whose help will be sought to identify participants. The assent will be read out to the participant and gatekeeper and their signature or left thumb print impression will be taken on the survey form.

This is to be mentioned that in the context of Bangladesh, the target population groups are highly stigmatised and their sexual behaviour (male-to-male sex) is not only culturally unaccepted but also prohibited by law so that there is no way we can identify and conduct face-to-face interview at their house where they live with their parents/siblings/other family members. Therefore, based on the context of Bangladesh, the process of taking consent and assent that is being followed in our study is being practised in conducing HIV surveillance rounds among KPs in Bangladesh over the years in the past.

3. We note that the grant information you provided in the ‘Funding Information’ and ‘Financial Disclosure’ sections do not match.

When you resubmit, please ensure that you provide the correct grant numbers for the awards you received for your study in the ‘Funding Information’ section.

Response: A separate funding statement is prepared to be submitted. Also, we have included funding information in the cover letter.

4. Thank you for stating the following in the Acknowledgments Section of your manuscript:

“The authors greatly thank the Department of Foreign Affairs, Trade and Development (DFATD), Canada for the funding of the study. The authors also thank the Institutional Review Board of icddr,b, for approving the study protocol. Icddr,b is also thankful to the Governments of Bangladesh, Canada, Sweden and the UK for providing core/unrestricted support to icddr,b.”

We note that you have provided funding information that is not currently declared in your Funding Statement. However, funding information should not appear in the Acknowledgments section or other areas of your manuscript. We will only publish funding information present in the Funding Statement section of the online submission form.

Please remove any funding-related text from the manuscript and let us know how you would like to update your Funding Statement. Currently, your Funding Statement reads as follows:

“The funders did not and will not have a role in study design, data collection and analysis, decision to publish, or preparation of the manuscript.”

Please include your amended statements within your cover letter; we will change the online submission form on your behalf.

Response: We have taken out the ‘Acknowledgement section” from the manuscript and provided that in the cover letter.

5. We note that you have indicated that data from this study are available upon request. PLOS only allows data to be available upon request if there are legal or ethical restrictions on sharing data publicly. For more information on unacceptable data access restrictions, please see <http://journals>.plos.org/plosone/s/data-availability#loc-unacceptable-data-access-restrictions.

**In your revised cover letter, please address the following prompts:**

a) If there are ethical or legal restrictions on sharing a de-identified data set, please explain them in detail (e.g., data contain potentially sensitive information, data are owned by a third-party organization, etc.) and who has imposed them (e.g., an ethics committee). Please also provide contact information for a data access committee, ethics committee, or other institutional body to which data requests may be sent. Please note that authors, including Corresponding Authors, are not permitted to be the sole point of contact for data requests.

b) If there are no restrictions, please provide the minimal anonymized data set necessary to replicate your study findings as either Supporting Information files or to a stable, public repository and provide us with the relevant URLs, DOIs, or accession numbers. For a list of acceptable repositories, please see <http://journals>.plos.org/plosone/s/data-availability#loc-recommended-repositories.

We will update your Data Availability statement on your behalf to reflect the information you provide.

Response: We did not use any data to prepare our manuscript therefore, these restrictions do not apply to our manuscript. However, the objectives of the proposed SRHR surveillance are to understand the burden, unmet needs and the overall situation of SRHR among MSM, MSW, transgender women (locally known as hijra) and FSW in selected areas in Bangladesh. This type of surveillance study, adopting a mixed-method, is being conducted for the first time not only in Bangladesh but also globally. We will collect data twice in year-1 (2022) and year-2 (2023). In the budget, we kept some money for publications where primary data from this surveillance study will be used to prepare manuscripts. We know that PLOS ONE publishes protocols that reach a variety of readers. Therefore, we thought that we also take an initiative to publish the protocol that may have huge impact in improving the wellbeing and the future design of SRHR services among the targeted population groups in other countries. This is to be noted that as soon as data are ready, we will start work to prepare manuscripts on various SRHR issues of the studied population groups for publications and we hope that we will submit manuscripts to the PLOS ONE.

6. Please include your full ethics statement in the ‘Methods’ section of your manuscript file. In your statement, please include the full name of the IRB or ethics committee who approved or waived your study, as well as whether or not you obtained informed written or verbal consent. If consent was waived for your study, please include this information in your statement as well.

Response: Our study was approved by the Research Review Committee (RRC) and Ethical Review Committee (ERC) of icddr,b. Mr. Shafiqul Alam Sarker, MD, Ph.D, FRCP is the Chairperson of RRC and Professor Ahmed Abu Saleh is the Chairperson of ERC. During submission, we had attached the approval copies from RRC and ERC.

7. We note that Figure 1  in your submission contain [map/satellite] images which may be copyrighted. All PLOS content is published under the Creative Commons Attribution License (CC BY 4.0), which means that the manuscript, images, and Supporting Information files will be freely available online, and any third party is permitted to access, download, copy, distribute, and use these materials in any way, even commercially, with proper attribution. For these reasons, we cannot publish previously copyrighted maps or satellite images created using proprietary data, such as Google software (Google Maps, Street View, and Earth). For more information, see our copyright guidelines: http://journals.plos.org/plosone/s/licenses-and-copyright.

We require you to either (1) present written permission from the copyright holder to publish these figures specifically under the CC BY 4.0 license, or (2) remove the figures from your submission:

a. You may seek permission from the original copyright holder of Figure 1  to publish the content specifically under the CC BY 4.0 license.

We recommend that you contact the original copyright holder with the Content Permission Form (http://journals.plos.org/plosone/s/file?id=7c09/content-permission-form.pdf) and the following text:

“I request permission for the open-access journal PLOS ONE to publish XXX under the Creative Commons Attribution License (CCAL) CC BY 4.0 (http://creativecommons.org/licenses/by/4.0/). Please be aware that this license allows unrestricted use and distribution, even commercially, by third parties. Please reply and provide explicit written permission to publish XXX under a CC BY license and complete the attached form.”

Please upload the completed Content Permission Form or other proof of granted permissions as an "Other" file with your submission.

In the figure caption of the copyrighted figure, please include the following text: “Reprinted from [ref] under a CC BY license, with permission from [name of publisher], original copyright [original copyright year].”

b. If you are unable to obtain permission from the original copyright holder to publish these figures under the CC BY 4.0 license or if the copyright holder’s requirements are incompatible with the CC BY 4.0 license, please either i) remove the figure or ii) supply a replacement figure that complies with the CC BY 4.0 license. Please check copyright information on all replacement figures and update the figure caption with source information. If applicable, please specify in the figure caption text when a figure is similar but not identical to the original image and is therefore for illustrative purposes only.

The following resources for replacing copyrighted map figures may be helpful:

USGS National Map Viewer (public domain): http://viewer.nationalmap.gov/viewer/

The Gateway to Astronaut Photography of Earth (public domain): http://eol.jsc.nasa.gov/sseop/clickmap/

Maps at the CIA (public domain): https://www.cia.gov/library/publications/the-world-factbook/index.html and https://www.cia.gov/library/publications/cia-maps-publications/index.html

NASA Earth Observatory (public domain): http://earthobservatory.nasa.gov/

Landsat: http://landsat.visibleearth.nasa.gov/

USGS EROS (Earth Resources Observatory and Science (EROS) Center) (public domain): http://eros.usgs.gov/#

Natural Earth (public domain): http://www.naturalearthdata.com/

Response: We have removed Figure-1 from the manuscript.

**Additional Editor Comments:**

Abstract:
- Minimize abbreviations in the abstract
Response: All abbreviations have been removed in the abstract.

Introduction and Methods,
- There are bulky paragraphs which needs paraphrasing like Line 112-124, 128-140, 263-276, 332-344, 376-386, 412-423, 443-454, 508-525

Response: We have taken care of these by rewriting the lines.

- The statements " Moreover, SRHR-related research has mainly focused on women, despite the importance of male sexual health. However, it is not possible to improve their overall health and quality of life if the issues related to SRHR of male and female KPs are not addressed properly and simultaneously' needs reference.

Response: The reference has been added in the manuscript.

- There is no clear description about the study design and data collection techniques for each specific objective.

Response: We have added Annex 5 to explain this. Please look at the file of All Annexes.

- Why did you used Finite Population Correction (FPC) for adjustment of sample size?
Response: Since the survey will be conducted without replacement from a finite population therefore, we followed using FPC in the sample size calculation as suggested by Leslie Kish (reference#23 in the manuscript).

- Minimize statistical formulas in the sample size calculation.
Response: Statistical formulas have been minimized in the manuscript.

[Note: HTML markup is below. Please do not edit.]

**Reviewers' comments:**

Reviewer's Responses to Questions

**Comments to the Author**

1. Does the manuscript provide a valid rationale for the proposed study, with clearly identified and justified research questions?

The research question outlined is expected to address a valid academic problem or topic and contribute to the base of knowledge in the field.

Reviewer #1: Yes

 2. Is the protocol technically sound and planned in a manner that will lead to a meaningful outcome and allow testing the stated hypotheses?

The manuscript should describe the methods in sufficient detail to prevent undisclosed flexibility in the experimental procedure or analysis pipeline, including sufficient outcome-neutral conditions (e.g. necessary controls, absence of floor or ceiling effects) to test the proposed hypotheses and a statistical power analysis where applicable. As there may be aspects of the methodology and analysis which can only be refined once the work is undertaken, authors should outline potential assumptions and explicitly describe what aspects of the proposed analyses, if any, are exploratory.

 Reviewer #1: Yes

 3. Is the methodology feasible and described in sufficient detail to allow the work to be replicable?

Descriptions of methods and materials in the protocol should be reported in sufficient detail for another researcher to reproduce all experiments and analyses. The protocol should describe the appropriate controls, sample size calculations, and replication needed to ensure that the data are robust and reproducible.

 Reviewer #1: Yes

 4. Have the authors described where all data underlying the findings will be made available when the study is complete?

The [PLOS Data policy](https://journals.plos.org/plosone/s/materials-and-software-sharing) requires authors to make all data underlying the findings described in their manuscript fully available without restriction, with rare exception, at the time of publication. The data should be provided as part of the manuscript or its supporting information, or deposited to a public repository. For example, in addition to summary statistics, the data points behind means, medians and variance measures should be available. If there are restrictions on publicly sharing data—e.g. participant privacy or use of data from a third party—those must be specified.

 Reviewer #1: No

 5. Is the manuscript presented in an intelligible fashion and written in standard English?

PLOS ONE does not copyedit accepted manuscripts, so the language in submitted articles must be clear, correct, and unambiguous. Any typographical or grammatical errors should be corrected at revision, so please note any specific errors here.

 Reviewer #1: No

 6. Review Comments to the Author

Please use the space provided to explain your answers to the questions above and, if applicable, provide comments about issues authors must address before this protocol can be accepted for publication. You may also include additional comments for the author, including concerns about research or publication ethics.

You may also provide optional suggestions and comments to authors that they might find helpful in planning their study.

(Please upload your review as an attachment if it exceeds 20,000 characters)

Reviewer #1: Abbreviations should not be used in the abstract.

Response: All abbreviations have been removed in the abstract.

Abstract: Line 28: ‘’SRHR’’ use the full form upon initial usage.

Response: Full form of SRHR is provided at the first usage.

Background: Line 77-78: “UN’’, ‘’UNFPA”, and “UNHCR’’ at first, use a full form.

Response: Full form of “UN’’, ‘’UNFPA”, and “UNHCR’’ is provided.

Background: Line 88, 215: It is not desirable to begin a paragraph with an abbreviation

Response: The line has been edited.

Background: Line 90: “UNHRC’’ At first, use a full form

Response: Full form of “UNHRC’’ is provided.

In the background section, there are long sentences from a single reference. Try to conduct a thorough review and integrate your paragraphs from different literature.

Response: This has been taken care of as you have suggested.

Your primary objective and the first secondary objective are the same. Why?

Response: The objectives have been edited. Thank you for the notification.

In the ethics statement: ‘’ The verbal consents of the participants were obtained’’. This statement is incorrect and should be corrected.

Response: The line has been edited.

Line 189, ‘’ Assent will be taken from those who will be 15 to less than 18 years of age’’ Are you going to take assent from the participant itself?

Response: Informed written assent will be taken from the participants of 15 to less than 18 years of age and informed written consent will be taken from those who will be 18 years or above years of age. Written assent will be taken from local gatekeepers (such as, hawkers in the spots or someone who belongs to the community of the participant and at the same time knows the participant very well and older in age, at least 18 years, than the participant) whose help will be sought to identify participants. The assent will be read out to the participant and gatekeeper and their signature or left thumb print impression will be taken on the survey form.

This is to be mentioned that in the context of Bangladesh, the target population groups are highly stigmatised and their sexual behaviour (male-to-male sex) is not only culturally unaccepted but also prohibited by law so that there is no way we can identify and conduct face-to-face interview at their house where they live with their parents/siblings/other family members. Therefore, based on the context of Bangladesh, the process of taking consent and assent that is being followed in our study is being practised in conducing HIV surveillance rounds among KPs in Bangladesh over the years in the past.

Avoid long paragraphs throughout the manuscript.

Response: This has been taken care of as you have suggested.

Be cautious about the usage of abbreviations throughout the manuscript.

Response: All abbreviations are checked and actions taken as suggested.

 7. PLOS authors have the option to publish the peer review history of their article ([what does this mean?](https://journals.plos.org/plosone/s/editorial-and-peer-review-process#loc-peer-review-history)). If published, this will include your full peer review and any attached files.


If you choose “no”, your identity will remain anonymous but your review may still be made public.
Response: We prefer to choose ‘yes’.

**Do you want your identity to be public for this peer review?** For information about this choice, including consent withdrawal, please see our [Privacy Policy](https://www.plos.org/privacy-policy).

 Reviewer #1: No[NOTE: If reviewer comments were submitted as an attachment file, they will be attached to this email and accessible via the submission site. Please log into your account, locate the manuscript record, and check for the action link "View Attachments". If this link does not appear, there are no attachment files.]

While revising your submission, please upload your figure files to the Preflight Analysis and Conversion Engine (PACE) digital diagnostic tool, <https://pacev2.apexcovantage.com/>. PACE helps ensure that figures meet PLOS requirements. To use PACE, you must first register as a user. Registration is free. Then, login and navigate to the UPLOAD tab, where you will find detailed instructions on how to use the tool. If you encounter any issues or have any questions when using PACE, please email PLOS at [figures@plos.org](mailto:figures@plos.org). Please note that Supporting Information files do not need this step.

*In compliance with data protection regulations, you may request that we remove your personal registration details at any time.* [*(Remove my information/details)*](https://www.editorialmanager.com/pone/login.asp?a=r)*. Please contact the publication office if you have any questions.*
